# Just a Joke? Adolescents’ Preferences for Humor in Media Entertainment and Real-Life Aggression

**DOI:** 10.1080/15213269.2022.2080710

**Published:** 2022-06-12

**Authors:** Amber van der Wal, J. Loes Pouwels, Jessica Taylor Piotrowski, Patti M. Valkenburg

**Affiliations:** Amsterdam School of Communication Research, University of Amsterdam, Amsterdam, The Netherlands

## Abstract

Humorous media entertainment frequently punctuates the everyday lives of adolescents. Theorists have suggested that this exposure may impact behavior, particularly real-life aggression. Specifically, exposure to prosocial (coping) humor in media entertainment is posited to decrease aggression, whereas the reverse has been argued for exposure to antisocial (disparaging and slapstick) humor. Despite these suppositions, little empirical evidence about this relationship exists. To fill this gap, this study employed a cohort-sequential design using latent growth curve models to estimate the (co-)development of adolescents’ preferences for television shows featuring disparaging, slapstick, and coping humor and aggression from age 10 to 17. Results showed that at the onset of adolescence, especially boys had a higher preference for shows with disparaging and slapstick humor than with coping humor. However, over the course of adolescence, boys’ and girls’ preferences for shows with coping humor increased, while especially girls’ preferences for shows with disparaging and slapstick humor decreased. These preferences were unrelated to adolescents’ aggression. Our findings provide an important addition to the ongoing media effects debate. Taken together, they offer room for optimism and point toward an increased focus on the potential positive rather than the negative sides of humor in the lives of young people.

Different age groups often have a distinctive taste in humor (Buijzen & Valkenburg, 2004). This is especially the case for adolescents, for whom humor binds and demarcates the peer group, which is reflected in adolescents’ dismissal of younger children’s taste in humor (e.g., “that’s stupid”) as well as in their use of humor to distance themselves from parents and other authority figures (Oppliger & Zillmann, 1997). Yet, little is known about the specific types of humor adolescents like, especially in media entertainment. Furthermore, although adolescents differ as a group from younger children and adults, their preferences likely develop *within* this age period as well. Specifically, due to increases in social intelligence and cognitive skills, adolescents may prefer more complex humor types over the course of adolescence (Fox, Hunter & Jones, 2016). In addition, boys and girls may develop different types of humor preferences in media entertainment, and these may change with age as well (Dowling, 2014). As such, a developmental perspective is needed to get insight into how boys’ and girls’ humor type preferences in media entertainment change over the course of adolescence.

As a first step, Van der Wal, Piotrowski, Fikkers and Valkenburg (2020) conducted a content analysis of 10- to 14-year old’s favorite television shows and found that these shows contained ten different types of humor, which varied in terms of content, cognitive structure, and prevalence. While all ten types are relevant from a media entertainment perspective, arguably, three humor types seem especially important in the context of adolescents’ development, as frequent exposure to these humor types in media entertainment may be positively or negatively associated with adolescents’ development of real-life aggression. The first two are aggressive (antisocial) in nature, namely disparaging (making fun of someone, ridiculing someone) and slapstick (physically aggressive, “pie in the face”) humor. The third is a prosocial humor type called coping humor, which functions as a coping mechanism to deal with life’s difficulties by joking about frustrations, hardships, and so on (Martin, Puhlik-Doris, Larsen, Gray & Weir, 2003).

For disparaging and slapstick humor, researchers have argued that frequent exposure to these humor types in media entertainment may sanction aggression in viewers (Bandura, 2001; Martins & Riddle, 2021; Potter & Warren, 1998). First, embedding aggression in humor may provide a moral disengagement cue, thereby increasing the likelihood of imitation (Martins, Mares, Malacane & Peebles, 2020). Second, disparaging and slapstick humor are often portrayed without negative consequences and are associated with social rewards (e.g., peer approval, making others laugh), which may also lower the threshold for imitation. Third, the combination of aggression and humor may trivialize the aggression (as it is “just a joke”) sending the message that aggressive behavior is acceptable (Potter & Warren, 1998). In contrast, media entertainment featuring coping humor shows the audience how to maintain a humorous perspective, even when faced with potentially stressful events and situations. As such, exposure to coping humor may minimize aggression by showing viewers a more functional way for tension release (Kuiper, 2012; McCullars et al., 2020). Yet, the relationship between adolescents’ preferences for media entertainment with disparaging, slapstick, and coping humor and aggression has not been empirically examined.

Given the gaps identified in the literature, the aim of this study is fivefold. First, we will examine how preferences for media entertainment featuring disparaging, slapstick, and coping humor develop over the course of adolescence. Second, we will examine how adolescents’ aggression develops across adolescence. Third, we will examine how initial levels of aggression are associated with initial levels of humor type preferences and how their subsequent developmental trajectories are related to each other. Fourth, we will examine whether adolescents’ preferences for entertainment featuring disparaging, slapstick, and coping humor are associated with subsequent changes in aggression. Fifth, we will explore how adolescents’ sex guides these associations. We address these goals by using a longitudinal accelerated design in which we link a longitudinal content analysis of adolescents’ preferences for media entertainment featuring disparaging, slapstick, and coping humor to longitudinal survey data on aggression from the same adolescents.

## Development of Humor Type Preferences from Early to Late Adolescence

The first goal of this study is to examine how preferences for media entertainment featuring coping, disparaging, and slapstick humor develop from early to late adolescence. There is little previous research on this topic. Yet, development level has been found to be a strong predictor of media use preferences (Valkenburg & Cantor, 2000). The “cognitive congruency principle” (Zigler, Levine & Gould, 1966) posits that this is because humor is considered the funniest when it is optimally challenging (i.e., not too easy and not too difficult). Consider examples of coping humor which require the cognitive flexibility to turn a difficult situation into something humorous (e.g., a person who lost his job joking about having all the time in the world). Or take an average episode of *House M.D*. which contains a lot of biting, sarcastic remarks and indirect mockery, all exemplars of disparaging humor, which are cognitively challenging to understand. For both humor types, one would expect a certain level of cognitive development is necessary to understand and appreciate the content. Thus, in line with the cognitive-congruency principle, it seems reasonable to anticipate that coping and disparaging humor become more popular with age (Fox, Hunter & Jones, 2016; Führ, 2002).

Compared to coping and disparaging humor, slapstick humor is considered simple in nature. Slapstick humor is seen in, for example, a show like *Tom and Jerry*, in which Tom keeps hitting Jerry on the head with a pan, with his eyes popping out at every hit. Slapstick can be seen as the humorous counterpart of media violence (i.e., physical aggression), characterized by “an exaggerated display of violence that is not accompanied by realistic consequences” (Jorgensen, Quist, Steck, Terry & Taylor, 2008, p. 27). Preference for entertainment featuring slapstick humor, when compared to coping or disparaging humor, seems to be less consistent with predictions of cognitive congruency. Specifically, despite slapstick’s simplicity, which makes it already popular among young children, it seems to be attractive (at least to a certain extent) among all age groups (Buijzen & Valkenburg, 2004; Jorgensen, Quist, Steck, Terry & Taylor, 2008). Even more, as previous research on the non-humorous counterpart of slapstick found an increase in adolescents’ preferences for physical aggression in media entertainment with age (Slater, Henry, Swaim & Anderson, 2003), we may also see adolescents’ preference for media entertainment featuring slapstick humor increase in adolescence. Therefore, we hypothesize:

**Hypothesis 1 (H1)**: Adolescents’ preferences for media entertainment featuring proportionally more (a) coping, (b) disparaging, and (c) slapstick humor increase from early to late adolescence.

## Development of Aggression from Early to Late Adolescence

Our second goal is to investigate whether adolescents’ initial levels of aggression are related to their initial humor type preferences and whether the subsequent developmental trajectories over time are related to each other (i.e., co-develop). In order to do so, we first have to examine the normative development of adolescents’ aggression from early to late adolescence. In previous research on aggression trajectories, although support has been found for heterogeneous pathways of aggression (e.g., small groups of adolescents scoring persistently high on aggression, or even keep increasing in level of aggression during adolescence, Cleverley, Szatmari, Vaillancourt, Boyle & Lipman, 2012), most adolescents seem to follow a moderate-declining trajectory into late adolescence (Cleverley, Szatmari, Vaillancourt, Boyle & Lipman, 2012). Specifically, studies reported a curvilinear trajectory from early to late adolescence, with a small increase in aggression from early to middle adolescence (usually between 13 and 15 years of age) followed by a subsequent decline (Cleverley, Szatmari, Vaillancourt, Boyle & Lipman, 2012; Karriker-Jaffe, Foshee, Ennett & Suchindran, 2008). This short increase has been explained by the “maturity gap,” which is experienced by young adolescents who feel mature biologically, but are dependent socially, and then act aggressively to signal maturity (Moffitt, 1993). As such, we hypothesize:

**Hypothesis 2 (H2)**: Adolescents’ aggression is expected to follow a curvilinear trajectory, with an increase in aggression from early to mid-adolescence, followed by a decline into late adolescence.

## The Co-Development of Aggression and Humor Type Preferences

Now that the separate development of adolescents’ humor type preferences and aggression have been discussed, we will focus on the relationship between the two. As research on adolescents’ selective exposure to *non-humorous* aggressive content has shown that aggressive adolescents tend to select more – both verbally and physically – aggressive media entertainment (Banerjee, Greene, Krcmar & Bagdasarov, 2009; Slater, Henry, Swaim & Anderson, 2003), an important open question is whether this is also the case for humorous aggressive content (i.e., disparaging and slapstick humor). A recent study suggests that this might be the case. Allen, Ash and Anderson (2021) found that dark triad personality traits like psychopathy (which includes antisocial behavior) were associated with enjoyment of a movie clip featuring aggressive humor. The authors suggest that related traits, such as an individual’s aggressive tendencies, might also be associated with liking humorous aggressive content (Allen, Ash & Anderson, 2021).

This aggression-induced preference of aggressive humor would make sense from a theoretical point of view. According to the benign violations theory (McGraw & Warren, 2010), humor occurs when a situation is simultaneously evaluated as a norm violation and as benign, which can occur in both slapstick (benign violations of personal dignity) or disparaging humor (benign violation of moral norms). These violations are a matter of perception – what is benign to some may be perceived as offensive (e.g., too aggressive) by others. In light of that, we would expect that familiarity with aggression may lead aggressive individuals to perceive aggressive humor types as a more *benign* norm violations and consequently as humorous. Specifically, we expect that adolescents’ initial levels of aggression will predict their preferences for media entertainment featuring proportionally more aggressive humor types. In addition, we expect that subsequent changes in their developmental trajectory of aggression will predict changes in their developmental trajectory of aggressive humor type preferences. For example, if an adolescent’s level of aggression decreases, we also expect the preference for media entertainment featuring aggressive humor types to decrease (called co-development). As such, we formulate the following hypotheses for disparaging and slapstick humor:

**Hypothesis 3 (H3)**: Adolescents’ initial levels of aggression will be positively related to initial levels of preference for media entertainment featuring proportionally more (a) disparaging and (b) slapstick humor.

**Hypothesis 4 (H4)**: The development of adolescents’ aggression will be positively related to the development of preferences for media entertainment featuring proportionally more (a) disparaging and (b) slapstick humor over time.

There is no research investigating how adolescents’ levels of aggression predict their preferences for media entertainment featuring coping humor. However, previous research has examined level of aggression in relation to the broader umbrella category of prosocial content. For example, Padilla-Walker, Coyne, Collier and Nielson (2015) measured adolescents’ aggression and preference for prosocial content in television shows two years apart and found no association between the two at either time points. So, neither initial levels of preference were related, nor were there indications of an association between adolescents’ aggression and preference for prosocial content later on (co-development). Given these patterns and the limited body of extant work, we formulate a research question for coping humor regarding these two types of associations:

**Research Question 1 (RQ1)**: How are (a) adolescents’ initial levels of aggression related to initial levels of preference for media entertainment featuring proportionally more coping humor and (b) how does the development of adolescents’ aggression relate to the development of their preferences for media entertainment featuring proportionally more coping humor over time?

## Adolescents’ Humor Type Preferences and Subsequent Changes in Aggression

The third goal of this study is to examine whether adolescents’ initial humor type preferences are associated with a *subsequent* change in aggression. Previous research has primarily focused on humor as a binary (either present or absent) contextual feature of media violence (i.e., non-humorous physical aggression), in which it has been characterized as a risk factor that may lower the threshold for adolescents to engage in real-life aggression (Martins & Riddle, 2021). We would argue that not only humorous physical aggression (i.e., slapstick) in media entertainment may be related to aggression, but also humor paired with indirect and verbal aggression (i.e., disparaging humor). Maybe even more so, because the threshold to engage in verbal and particularly indirect aggression is lower than for physical aggression, and the ramifications are smaller (Card, Stucky, Sawalani & Little, 2008). This notion seems supported by a recent study conducted in the context of tween sitcoms. Specifically, Martins, Mares, Malacane and Peebles (2020) found that tweens who perceived aggression in sitcoms as funny were more likely to imitate the portrayed aggression.

In contrast to the two aggressive humor types, exposure to coping humor in media entertainment may prevent adolescents from lashing out and hence, a preference for coping humor may be associated with a subsequent decrease in aggression. This notion seems supported by previous research on non-humorous prosocial content on television, in which preference for viewing prosocial content at the initial time point was negatively associated with aggression two years later (Padilla-Walker, Coyne, Collier & Nielson, 2015), which raises the expectation that the same may be the case for frequent exposure to coping humor. As such, we hypothesize:

**Hypothesis 5 (H5)**: High initial levels of preference for media entertainment featuring proportionally more (a) disparaging and (b) slapstick humor will be associated with an increase in aggression over time, whereas (c) high initial levels of preference for media entertainment featuring proportionally more coping humor will be associated with a decrease in aggression over time.

## The Role of Sex in the (Co-)development of Aggression and Humor Type Preferences

The final goal of this study is to explore the role of sex in the (co-)development of aggression and humor type preferences, as there are indications that there may be sex differences in both. For example, research on interpersonal humor found that girls prefer coping humor, while boys seem to prefer slapstick humor (Führ, 2002; Jorgensen, Quist, Steck, Terry & Taylor, 2008). These differences may become more prominent with age as differences in entertainment preferences between boys and girls generally become bigger in adolescence (Calvert & Wilson, 2010). Furthermore, with regard to adolescents’ development of aggression, studies have showed that boys consistently display a higher level of physical and verbal aggression than girls, but boys and girls seem to follow parallel developmental trajectories across adolescence (Cleverley, Szatmari, Vaillancourt, Boyle & Lipman, 2012; Karriker-Jaffe, Foshee, Ennett & Suchindran, 2008). Based on these sex differences, there may also be sex differences in the co-development of aggression and humor type preferences in media entertainment. However, as there is no existing research on this topic, we will explore sex differences by posing the following research question:

**Research Question 2 (RQ2)**: What is the role of adolescents’ sex in the relation between adolescents’ aggression and their preferences for media entertainment featuring disparaging, slapstick, and coping humor from early to late adolescence?

## Method

We preregistered our study using the AsPredicted-template on the Open Science Framework (see, Van der Wal, Pouwels, Piotrowski & Valkenburg, 2021).

### Participants

This study is based on longitudinal data collected from 165 adolescent participants (52.1% girls) from five age cohorts: 10-year-olds (*n* = 34), 11-year-olds (*n* = 29), 12-year-olds (*n* = 37), 13-year-olds (*n* = 35), and 14-year-olds (*n* = 30). This sample is part of a larger project of which the complete process is detailed in Van der Wal, Piotrowski, Fikkers and Valkenburg (2020). Conducting an a-priori power analysis proved difficult because the method used in this study is both unprecedented and quite complex. Therefore, a simulation study was conducted under the auspices of a methodologist to ensure that our sample size of 165 adolescents was sufficient. Ethical approval was secured for the project, and all participants were required to provide informed consent. Relevant to the current analysis, adolescents were followed for four consecutive years between fall of 2012 and fall of 2015. Each year, participants filled in a survey in which both an assessment of their media habits and aggression was included.

### Measures

#### Aggression

All participants completed the Direct and Indirect Aggression Scale (DIAS; Björkqvist, Lagerspetz & Österman, 1992) each year (4 waves). The DIAS is frequently used to measure adolescents’ aggression and capable of detecting change over time (Collett, Ohan & Myers, 2003; Wallenius & Punamäki, 2008). The DIAS consists of 14 items that measure how often adolescents, when angry with another peer, use physical (e.g., hitting or pushing someone), verbal (e.g., insulting or threatening someone), and indirect aggression (e.g., gossiping behind someone’s back, trying to get others to dislike the person). Items were answered on a five-point Likert scale ranging from 1 (= *never*) to 5 (= *very often*). For this study, we created one composite score of aggression per wave by computing the average score of all 14 items. For Wave 1–4, Cronbach’s alpha ranged between .88 and .93.

#### Adolescents’ Favorite Shows

On each measurement occasion, adolescents listed up to three favorite television shows available via television, streaming platforms, or DVD (cf., Manganello & Chauhan, 2011). Each of these shows were coded for their target audience: children (15.8%), t(w)eens (10.8%), or mature/general audience (73.4%). The favorite television shows listed by adolescents in the survey formed the basis for a content analysis to discern humor type preferences. The television shows listed in Wave 1 (*n* = 107) had already been coded as part of a previous study (Van der Wal, Piotrowski, 2020). For this study, we also coded the television shows adolescents had listed as their favorite in Wave 2, 3, and 4, based on the same procedure used for Wave 1. This resulted in a coding sample of 134 shows (Wave 2: 45 shows, Wave 3: 50 shows, Wave 4: 39 shows).

As with Wave 1, for each show, two episodes were included in the content analysis (cf., Banerjee, Greene, Krcmar & Bagdasarov, 2009). Episodes were randomly selected from the season that aired around the time of the survey. In total, 268 episodes were included, ranging from approximately ten minutes to an hour and a half in length, with a mean duration of 38 minutes and 17 seconds. All episodes were then divided into scenes, which served as the coding unit. A scene was defined as “an uninterrupted sequence of thematically-related activities occurring within a given physical context” (Weaver, 1991). The coding sample consisted of 7,144 scenes.

The first author (who also participated in the coding) trained coders extensively, after which intercoder reliability was assessed using Krippendorff’s alpha (Lombard, Snyder‐Duch & Bracken, 2002). Sufficient intercoder reliability was reached (Krippendorff’s alpha being .80 or higher). Next, the 134 television shows were randomly assigned among coders. Coders first had to watch a show without coding to get familiar with it and then again to code each scene for the presence of coping, disparaging, and slapstick humor. See also the coding manual. We did not code the scenes for non-humorous aggression because an earlier study by Van der Wal, Fikkers and Valkenburg (2020) yielded correlations of non-humorous aggression with disparaging and slapstick humor around zero (i.e., r = .03 and r = .01 respectively).

After combining the data of Wave 1 with that of Wave 2–4, the total content-analytic sample consisted of 12,920 scenes, with dichotomous scores for the three humor types in each scene. Next, we summed the number of scenes within an episode that featured coping, disparaging, and/or slapstick humor and then for the two episodes coded per television show. Because a higher number of scenes means a higher potential frequency of the three humor types, we needed to account for variation in the number of scenes. Therefore, we created proportion scores reflecting the proportion of scenes featuring coping, disparaging, and slapstick humor relative to the total number of scenes in that adolescent’s favorite television show(s). For example, Adolescent X listed a show with 30 scenes as his first favorite show and a show with 36 scenes as his second favorite. In the first show, disparaging humor was coded as present in six scenes and in the second show in 12 scenes. This means 18/66 * 100 = 27.3% as that individual’s score for disparaging humor. This was done for all four waves. These scores functioned as the operationalization of adolescents’ humor type preferences. Proportion scores on a show-level (by genre) are presented as Supplement A.

### Analytic Approach

We analyzed the data according to our preregistered analysis plan. To test our hypotheses and research questions regarding the (co-)development of adolescents’ aggression and humor type preferences across adolescence, in line with recommendations by Keijsers and van Roekel (2019), we used multiple-group Latent Growth Curve Models (LGCMs) with an accelerated design in Mplus Version 8 (Muthén & Muthén, 1998–2017). In these models, development was expressed by an intercept and a slope factor. We preregistered that the intercept would correspond with initial levels of aggression and humor type preferences at age 10 and incorporated the initial levels in the hypotheses. However, to avoid multi-collinearity between the intercept and slopes (Wainer, 2000), we deviated slightly from the preregistration by centering the intercept at the midpoint of the age range (i.e., age 13.5) instead of age 10. Consequently, rather than at the initial levels, we now tested the correlation between variables at the midpoint of the age range (i.e., age 13.5; see Supplement B for a graphical representation). We analyzed the data in several steps.

We first examined whether the different cohorts were sufficiently similar to be combined into one accelerated (cohort-sequential) growth model. To that end, we compared constrained models in which the intercepts and slopes were constrained across cohorts with unconstrained models in which the intercept and slopes were estimated freely across cohorts. As preregistered, because all variables were highly skewed, we used maximum likelihood robust estimation (Kline, 2011). As this method insufficiently controlled for skewness (indicated by an insufficient model fit, see Supplement C), we log transformed the data (Tabachnick, Fidell & Ullman, 2013). After the log transformation, comparison of the constrained and unconstrained models showed that the fit of the constrained models did not differ substantially from the unconstrained version of the models for aggression, disparaging, and slapstick humor, meaning we could proceed with the cohort-sequential approach. For coping humor, we needed to further respecify the model by freely estimating the intercept in each group, given that mean levels of coping humor at age 13.5 varied between the cohorts (see Supplement D).

Second, we examined the development of aggression and humor preferences from age 10 to 17 by estimating four linear multiple-group LGCMs. This approach leads to missing data by design. We controlled for missing data patterns by using full information maximum likelihood estimation as the data were missing completely at random. Third, we compared linear models to quadratic models to examine whether the development was linear or curvilinear in nature. Adding a quadratic slope led to complex errors. We therefore constrained the quadratic slope variance to zero and as a result, all models ran without errors, except for coping humor, which led us to conclude that estimating a model with a quadratic slope was too complex for coping humor. After determining the best model for each variable, we added sex to the model to explore the role of sex (RQ2). Finally, in our preregistration we stated that we would explore potential differences in subtypes of aggression (physical, verbal, indirect). However, correlations between the subtypes of aggression were so high (r = .69-.85) that we decided not to pursue this.

As a fourth and final step, we examined how the development of aggression was related to the development of coping, disparaging, and slapstick humor (RQ1, H3-H5), by estimating three Parallel Process Models (PPM, see Supplement E). In each of these parallel LCGMs, we estimated the development of aggression and one humor type preference together in one model. Each construct had its own intercept and slopes. See Supplement B for a graphical representation.

In step 1 to 4, we evaluated the fit of the models according to preregistered criteria. Nested models (i.e., constrained vs. unconstrained models; linear vs. quadratic models) were compared with the Satorra Bentler Chi-Square Test (significance indicates relative better model fit) combined with the Akaike information criterion (AIC) and Bayesian Information Criterion (BIC). Lower AIC and BIC values indicate better fit of the model (Geiser, 2012). When the AIC, BIC and Chi-square did not correspond, the Chi-Square test was leading in our assessment. Absolute model fit was evaluated using the following indices: the Root Mean Square Error of Approximation (RMSEA), the Comparative Fit Index (CFI), and the Tucker Lewis Index (TLI). Two out of three criteria needed to hold: RMSEA < .08; CFI > .90, TLI > .90 (McDonald & Ho, 2002). When model fit was insufficient or did not converge, models were respecified according to our preregistered approach (see Supplement C).

## Results

### Descriptive Statistics

Descriptives for aggression and the three humor type preferences at each measurement occasion are presented as Supplement F. The mean aggression scores were fairly low (ranging from 1.72 in Wave 1 to 1.57 in Wave 4 on a 5-point scale) and correlated strongly with each other at subsequent waves (*r* = .45 to .60). Furthermore, across measurement occasions, adolescents preference scores were the highest for shows featuring disparaging humor (ranging from 28.2% of scenes in adolescents’ favorite shows in Wave 1 to 21.3% in Wave 4), followed by slapstick humor (ranging from 15.1% of scenes in adolescents’ favorite shows in Wave 1 to 8.4% in Wave 4), and then coping humor (ranging from 3.8% of scenes in Wave 1 to 5.7% in Wave 4).

Adolescents’ preference scores for shows featuring disparaging humor at each subsequent wave were significantly correlated with each other (*r* = .26 to .27), as were the subsequent slapstick (*r* = .35 to .40) and coping humor scores (*r* = .18 to .30). In addition, at each wave, disparaging and slapstick humor were significantly positively correlated (*r* = .58 to .71), whereas disparaging and coping humor were significantly positively correlated at three (*r* = .25 to .32) out of four waves. Slapstick and coping humor correlated significantly negatively at Wave 1 (*r* = −.35) and non-significantly in the other waves.

### Developmental Trends

#### Coping Humor

**Figure 1.**
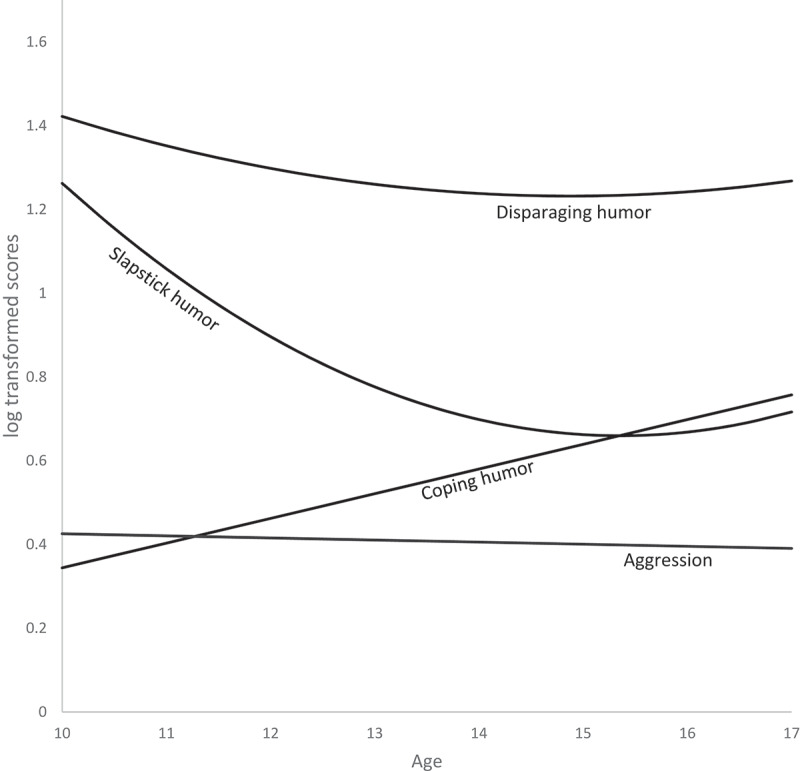
Estimated Development of Adolescents’ Aggression and Humor Type Preferences From Age 10 to 17.

Note. For ease of interpretation, we used the estimated mean intercept for coping humor in the figure. In Supplement D detailed depictions of the intercepts of each cohort can be found.

For coping humor, we hypothesized that adolescents’ preference for television shows featuring coping humor would increase from early to late adolescence (H1a). Estimating a linear growth model resulted in good absolute model fit (while the quadratic model led to errors) and a significant positive slope (see, Table 1). This indicates that there was a significant linear increase in adolescents’ preference for television shows featuring coping humor from age 10 to 17 (see, Figure 1), which supports the hypothesis. In addition, the variances for the intercept (σ = 0.05, *p* = .02) and slope (σ = 0.02, *p* = .02) were significant, suggesting some individual differences in both the level of and rate of change in preference for coping humor.

**Table 1.** Development of Aggression and Humor Type Preferences.

	Coping humor		Disparaging humor		Slapstick humor		Aggression	
	Linear	Quadratic^b^	Linear	Quadratic	Linear	Quadratic	Linear	Quadratic
Intercept	0.45–0.62 ^a^**		1.27**	1.25**	0.79**	0.73**	0.41**	0.41**
Linear Slope	0.06*		−0.02*	−0.02*	−0.08**	−0.08**	−0.005	−0.004
*d*	0.28		−0.12	−0.10	−0.31	−0.30	−0.07	−0.07
Quadratic Slope	NA		NA	0.008*	NA	0.02**	NA	−0.002
*d*	NA		NA	0.04	NA	0.08	NA	−0.03
Absolute fit indices								
RMSEA	0.05		0	0	0.06	0	0.10	0.1
CFI	0.90		1	1	0.90	1	0.91	0.92
TLI	0.92		1.04	1.14	0.93	1.09	0.93	0.94
Relative fit indices								
AIC	692.33		533.13	530.98	874.74	863.16	−1243.69	−1244.41
BIC	794.83		623.20	624.16	952.39	943.91	−1153.62	−1151.22
Deviance	626.33		475.13	470.98	824.74	811.16	−1301.69	−1304.41
Likelihood Ratiotest χ^2^(df) Significance level *p*				4.47(.93) .03*		9.70(1.40) .002*		2.27(1.20) .13

Note. Aggression and humor scores have been log transformed. Development is expressed in three growth factors: intercept (mean level at midpoint of the age range), linear slope (i.e., linear increase or decrease), and quadratic slope (i.e., curvature). Absolute model fit indicated by RMSEA = Root Mean Square Error of Approximation; CFI = Comparative Fit Index; and TLI = Tucker-Lewis Index. Relative model fit indicated by Satorra Bentler Chi-Square Test (significance indicates relative better model fit), combined with the Akaike information criterion (AIC) and Bayesian Information Criterion (BIC). *d* = approximate Cohen’s d effect size for the slope.

^a^For coping humor, the models were respecified by freely estimating the intercept in each group, because it varied between the cohorts (see Supplement D). This table included the range of the intercepts here.

^b^The quadratic model for coping humor resulted in errors and was deemed too complex. Relative fit of quadratic model was significantly better for disparaging and slapstick humor. * *p* < .05, ** *p* < .001.

#### Disparaging Humor

The hypothesis (H1b) was that adolescents’ preference for television shows featuring disparaging humor would increase from early to late adolescence. We tested this by estimating a linear and quadratic growth model for the development of adolescents’ preference for disparaging humor. Both models had an adequate absolute model fit. Yet, the relative fit of the quadratic model was significantly better than that of the linear model, S-Bχ^2^ (1) = 4.47, *p* = .03. As shown in Figure 1, disparaging humor followed a curvilinear development over time and decreased slightly from age 10 to 14 after which it increased again slightly, as indicated by a significant negative linear slope and positive quadratic slope (see Table 1). So, H1b was rejected. Furthermore, the intercept variance (σ = 0.04, *p* = .003) was significant, indicating individual differences in level of preference for disparaging humor.

#### Slapstick Humor

We hypothesized that adolescents’ preference for television shows featuring slapstick humor would increase from early to late adolescence (H1c). Similar to disparaging humor, both the linear and quadratic growth models had good absolute model fit, yet, the relative fit of the quadratic model was significantly better than the relative fit of the linear model, S-Bχ^2^ (1) = 9.70, *p* = .002. The quadratic model had a significantly negative linear slope and a significantly positive quadratic slope (see Table 1). As shown in Figure 1, this means that adolescents’ preference for slapstick humor in their favorite television shows followed a curvilinear decline over time. Specifically, slapstick humor decreased from age 10 to 15 after which it increased slightly again until age 17. This means that H1c was not supported. Again, the intercept variance (σ = 0.07, *p* < .001) was significant and therefore suggestive of individual differences in adolescents’ preference for slapstick humor.

#### Aggression

For adolescents’ aggression we expected to see a curvilinear trajectory, with an increase in aggression from early to mid-adolescence, followed by a decline (H2). Contrary to our expectation, the linear fit of the aggression model was good (see Table 1) and did not differ significantly from the quadratic model, S-Bχ^2^ (1) = 2.27, *p* = .13, indicating that aggression did not develop in a curvilinear way. Instead, there was a slight linear decrease (*p* = .056) in adolescents’ development of aggression from age 10 to 17 (see Figure 1). Furthermore, the variances for the intercept (σ = 0.01, *p* < .001) and slope (σ = 0.001, *p* = .006) were significant, which points at individual differences in both the mean level and development of aggression.

### Co-Development

To examine how the development of aggression is related to the development of disparaging, slapstick, and coping humor (RQ1, H3-H5), we estimated three Parallel Process Models (PPM), see Supplement B for a graphical representation. As can be seen in Table 2, none of the correlations related to H3-H5 and RQ1 were significant. Thus, mean levels and developmental trajectory of adolescents’ aggression were not related to mean levels and developmental trajectory of each of the three humor types, thereby rejecting H3-H5.

**Table 2.** Associations Between the Intercept and Slope of Aggression and Humor Type Preferences.

	Intercept Aggression *r*	Linear Slope Aggression *r*
Intercept Disparaging Humor	0.08 (H3a)	0.03 (H5a)
Linear Slope Disparaging Humor	0.08	0.12 (H4a)
Intercept Slapstick Humor	0.19 (H3b)	−0.07 (H5b)
Linear Slope Slapstick Humor	0.10	−0.22 (H4b)
Intercept Coping Humor	−0.11 (RQ1a)	0.05 (H5c)
Linear Slope Coping Humor	−0.36*	−0.14 (RQ1b)

Note. *r* = correlation. PPM’s are based on univariate LCGM’s. For aggression and coping humor, these models only included a linear slope. For disparaging and slapstick humor, models also included a quadratic slope. However, as we constrained the quadratic slope variance to zero, only the linear slopes of the humor preferences could be associated with the intercept and slope of aggression. Model fit of all PPM’s was slightly below adequate which may be due to the limited power of these models. However, because the univariate growth models had a good fit, we interpreted the results with caution.

* *p* = .03

### The Role of Sex

**Figure 2.**
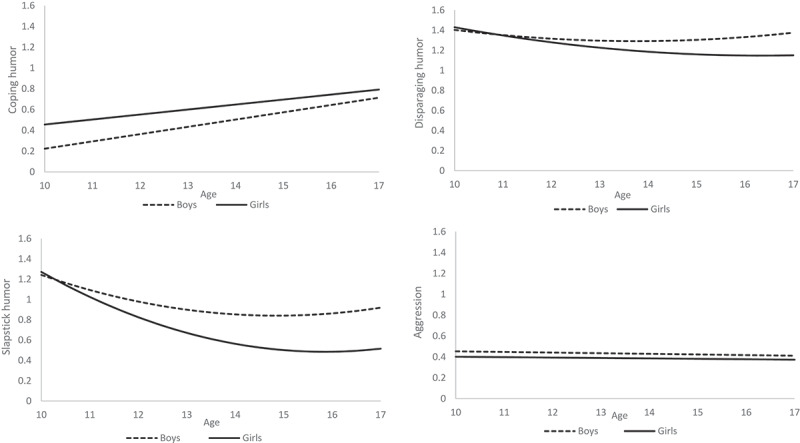
**a-d**. *Estimated Sex Differences in Development of Humor Type Preferences and Aggression.*

Note. For Figure 2a-d, the log transformed scores have been used

As the intercept and slope variances pointed at significant differences between adolescents in the levels and rate of change in aggression and humor type preferences, we explored whether adolescents’ sex (RQ2) could explain these differences by estimating conditional growth models. Results showed that there were significant sex differences in adolescents’ levels of aggression, levels of all three humor type preferences, and in the development of slapstick and disparaging humor. As shown in Figure 2a-d, girls’ level of aggression (b = −0.05, *p* < .001) and preference for television shows featuring disparaging (b = −0.09, *p* = .08) and slapstick humor (b = −0.26, *p* < .001) was lower than that of boys, whereas girls had a higher preference for television shows featuring coping humor (b = 0.16, *p* = .001). For television shows featuring disparaging humor, boys’ preference remained stable from age 10 to 17, while girls showed a significant decrease (b = −0.04, *p* = .05). Furthermore, while both boys and girls showed a decrease in preference for television shows featuring slapstick humor, girls showed a larger decrease in preference than boys (b = −0.06, *p* = .02). Finally, as preregistered, we also aimed to examine the role of sex in the Parallel Process Models. However, by adding sex to the models they no longer converged and yielded several errors. We therefore discontinued this exploration.

## Discussion

The present study enhanced our understanding of adolescents’ preferences for media entertainment featuring different humor types and real-life aggression in two ways. First, we found that at the onset of adolescence, especially boys showed a higher preference for television shows that featured a relative high amount of disparaging and slapstick humor and little coping humor. However, over the course of adolescence, boys’ and girls’ preferences for shows with coping humor increased, while especially girls’ preferences for shows with disparaging and slapstick humor decreased. Second, we revealed that these developmental trajectories of different humor type preferences were unrelated to adolescents’ level and developmental course of aggression.

### Explaining the Development of Adolescents’ Humor Type Preferences

Understanding coping humor requires the cognitive ability to change perspective in order to positively reappraise a threatening or depressing situation (Greve, Hauser & Rühs, 2021). This ability only starts to develop in adolescence. Therefore, we hypothesized that as adolescents mature, they would grow in their liking of shows featuring coping humor. And indeed, we found that at the onset of adolescence, adolescents’ favorite shows contained hardly any coping humor, yet with age, coping was featured more and more in adolescents’ favorite shows, even passing slapstick in frequency around age 15. Our findings also revealed that at age ten, girls’ preference for shows featuring coping humor was much higher than boys’. This could be explained by Lynn’s (1998) finding that from around the age of eight, girls mature cognitively faster than boys. These maturational sex differences usually start to level out from the age of 13–14 years (Lynn, 1998), which in turn corresponds with our finding that with age, boys’ and girls’ preference for shows featuring coping humor grew closer together.

In contrast to coping humor, at the onset of adolescence, the favorite television shows from the adolescents in our sample featured a lot of disparaging and slapstick humor. Contrary to expectation, however, this was followed by a decrease in adolescents’ preferences for shows featuring a lot of disparaging and slapstick humor with age, especially among girls. How could this be explained? It may be that personality characteristics, such as adolescents’ levels of sympathy (i.e., feelings of concern for another person) play a role here. For example, adolescents with higher levels of sympathy may dislike aggressive types of humor because they feel concern for the victim of the joke. For both boys and girls, sympathy levels increase from early to late adolescence (Vossen & Fikkers, 2021), with girls typically showing a steeper increase than boys (Carlo, Padilla-Walker & Nielson, 2015). This could explain both the general decrease in preference for shows with aggressive humor types with age and the steeper decrease for girls.

Taken together, there definitely seems to be a change in the composition of adolescents’ favorite shows with maturation. Of course, we cannot claim this is solely due to the types of humor featured in these shows, as there are other important program characteristics to consider (e.g., age-appropriateness, fantasy vs. reality). Inasmuch, experimental research is needed to disentangle the various factors related to adolescents’ developmentally-guided preferences. Furthermore, we explained this study’s findings through differences in level of cognitive development and sympathy. Yet, the adolescent period signifies many biological, physical, and personality changes that may affect their humor preferences. As such, it is important to determine what exactly causes their preferences. Therefore, future research is warranted to examine the underlying mechanisms of developmental changes in humor preferences, for example, by using Random-Intercept Cross-Lagged Panel Models.

### The Relation between Adolescents’ Humor Type Preferences and Aggression

Guided by the benign violations theory (McGraw & Warren, 2010), we expected that aggressive adolescents would perceive disparaging and slapstick humor as more benign and thus more humorous than non-aggressive adolescents. Yet, we found no relationship between adolescents’ level of aggression and their preference for television shows featuring aggressive humor types. In addition, we tested the theoretical assumption that those adolescents who are more frequently exposed to aggressive humor types would be more likely to increase in aggression over the course of adolescence, whereas those who are more frequently exposed to coping humor would be more likely to decrease in aggression (Kuiper, 2012; Potter & Warren, 1998). As adolescents’ preferences for the different humor types were unrelated to their developmental course of aggression, this assumption was not supported.

The null effects in the current study are in contradiction with previous work by Martins, Mares, Malacane and Peebles (2020), who found that liking of humorous aggression in sitcoms increased the likelihood of imitation. However, Martins et al. used hypothetical questions about the possibility that adolescents would engage in future aggressive acts. Yet, as the authors pointed out themselves, studies focusing on actual aggression found no effect of funny cartoon violence on children (e.g., Hapkiewicz & Stone, 1974), while work focusing on hypothetical aggression did find effects (e.g., Nathanson & Cantor, 2000). Our results are based on actual aggression and thus comparable to earlier research on humorous media violence using similar measures that found null effects. Furthermore, the null findings in our preregistered study seem to be supported by recent preregistered studies on non-humorous aggression (Drummond, Sauer & Ferguson, 2020; Ferguson, 2020). As there are hardly any studies on the relationship between exposure to humorous aggression in media entertainment and subsequent aggression, we hope that by making all our materials and analyses publicly available, this study offers a foundation for future research.

Our study design did not allow us to examine causal relationships, so some caution in interpreting our (lack of) findings is warranted. Moreover, in our sample – and in the Netherlands as a whole – adolescents generally score low on aggression. As such, it would be interesting to replicate our design in other countries and cultures where aggression is more varied. Another avenue for future research is to investigate which adolescents may and may not be susceptible to the effects of humorous aggression in media entertainment. Adolescents are a heterogeneous group and looking at aggregate levels as we did may have masked potential positive and/or negative effects for some. Unfortunately, a lack of statistical power precluded examining potential moderators and the variance around the overall effects. Future research with a larger sample size could benefit from using Latent Class Growth Models to examine whether different subgroups follow different developmental trajectories. Such an approach may also circumvent issues with skewed data, which may avoid the need to log transform the data and consequently simplify the interpretation of results.

### Looking Ahead

Although analyzing the content of favorite media entertainment as an operationalization of someone’s preference for certain media content has been an often-validated and recommended measure (e.g., Knobloch-Westerwick, 2014), we could not verify that adolescents listed a particular show as their favorite *because* of the humor in it. At the same time, humor was highly prevalent in all genres in our sample, is often listed as media entertainment’s most attractive characteristic, and can be a big turn-off when not liked (Valkenburg & Cantor, 2000). Inasmuch, while possibly not the only reason for selection, we feel confident that the type of humor in an adolescent’s favorite show at least plays a significant role. Future research could complement our use of favorites by adopting a reception-based approach in which the to-be-coded humorous content could be selected based on indicators of perceived funniness by the users (e.g., content on YouTube or social media platforms to which they respond with laughing emoji’s or phrases like “haha” and “lol”).

Studying humor is also challenging because it is labor-intensive. To illustrate, for our sample of 165 adolescents, we had to code 241 television shows, which consisted of nearly 13,000 scenes. Unless researchers have unlimited funding or time, this automatically means they are somewhat constrained in the number of participants they can include, which, as a result, also limits the power needed for several sophisticated methods of analyses (e.g., the cohort-sequential latent growth curve modeling design used in this study). As AI-based coding of humor is still in its infancy, the field needs to consider other ways to make the coding process more doable. Luckily, recent media entertainment phenomena such as TikTok and satirical Instagram accounts that are extremely popular among adolescents and young adults offer some possibilities in this respect, because content on these platforms is usually much shorter in duration and therefore less labor-intensive to code compared to television shows.

In addition, in looking ahead, our findings on the increasing popularity of television shows featuring proportionally more coping humor with age – and the absence of an association between humor preferences and aggression – emphasize the need for a more positive perspective on humor in media entertainment. This has become especially relevant during the COVID-19 pandemic, in which adolescents had to fall back on (social) media for information, entertainment, and social interaction. In the first two months of the pandemic, nearly half of TikTok videos tagged with #coronavirus were humorous, increasing to 68% in the third month, as insecurity and fear grew larger (Southwick et al., 2021). This suggests that humor in the face of adversity is an important coping mechanism, especially for adolescents. Furthermore, it indicates that humor may be an important tool in engaging and educating adolescents on topics of public health. While more research is needed on the specific circumstances in which humor in media entertainment can facilitate learning amongst adolescents, the present study suggests that understanding the benefits of coping humor would be a valuable addition to the field.

## Conclusion

This study employed a novel approach in which we used latent growth curve models with a cohort-sequential design to estimate the (co-)development of adolescents’ preferences for television shows featuring different coping, disparaging, and slapstick humor and aggression from age 10 to 17. This approach has provided valuable insights. Results showed that at the onset of adolescence, television shows featuring disparaging and slapstick humor are very popular, while television shows featuring coping humor are not. Yet, with age, adolescents’ preference for shows featuring aggressive humor types decreases, whereas their preference for shows featuring coping humor increases. Furthermore, adolescents’ level of aggression declines slightly and is not related to their humor type preferences. Although further research is needed into the exact relationship between the development of adolescents’ preferences for different humor types in media entertainment and aggression, this study’s findings are promising and point toward a future focus on the positive effects of humor, rather than the negative.

## Supplementary Material

Supplemental Material

## Data Availability

The data described in this article are openly available in the Open Science Framework at https://doi.org/10.17605/OSF.IO/GA6WC.

## Notes

Time-stamped preregistration can be found on https://doi.org/10.17605/OSF.IO/VPBNA

Materials, data and syntax files can be found on https://doi.org/10.17605/OSF.IO/GA6WC

## Open Scholarship

This article has earned the Center for Open Science badges for Open Data, Open Materials and Preregistered. The data and materials are openly accessible at https://doi.org/10.17605/OSF.IO/GA6WC and https://doi.org/10.17605/OSF.IO/VPBNA.
