# Supplementary material for: Just a Joke? Adolescents’ Preferences for Humor in Media Entertainment and Real-Life Aggression
Source: Media Psychol. 2022 Jun 12;25(6):797–813. doi: 10.1080/15213269.2022.2080710 (PMC9621101; doi:10.1080/15213269.2022.2080710)
Supplement: Supplemental Material [file HMEP_A_2080710_SM4645.docx]

**Supplement A
Characteristics of Sample of Television Shows by Genre**

| **Genre** | **% of shows^1^** | **% of humor- scenes^2^** | **% Disparaging humor (*SD*)** | **% Slapstick Humor (*SD*)** | **% Coping Humor (*SD*)** |
| --- | --- | --- | --- | --- | --- |
| Drama | 19.1 | 35.0 | 16.9 (12.2) | 4.2 (7.4) | 4.5 (6.1) |
| Action-adventure | 14.5 | 44.7 | 16.6 (13.9) | 7.2 (17.6) | 4.3 (6.5) |
| Thriller-Mystery | 10.4 | 30.5 | 13.3 (12.5) | 2.5 (5.9) | 4.5 (7.0) |
| Comedy | 25.7 | 95.2 | 50.2 (28.3) | 34.4 (24.1) | 7.1 (11.8) |
| Reality | 33.2 | 53.9 | 23.1 (19.7) | 10.3 (20.0) | 6.8 (11.9) |
| Talk show | 2.1 | 72.8 | 29.6 (15.3) | 10.3 (9.5) | 7.3 (29.8) |
| Edutainment | 10.8 | 40.0 | 11.44 (17.9) | 7.1 (11.2) | 3.5 (10.9) |

^1^Adds up to more than 100% because some shows fit into more than one multiple genre

^2^ Percentage of scenes in each genre that contains at least one type of humor

^3^ Percentage of scenes in each specific genre that contain disparaging, slapstick, or coping humor

**Supplement B**

**Visual Presentation of How Hypotheses and Research Questions Were Investigated**

- **For H3a, H3b, and RQ1a,** we wanted to know whether adolescents’ level of aggression is related to higher or lower preferences for television shows featuring disparaging, slapstick, and coping humor. We tested this by examining the correlation between the intercept for aggression and the intercept for each humor type preference.
- **For H4a, H4b, and RQ1b,** we wanted to know how the developmental trajectories of aggression and each humor type preference are related. For instance, intercepts may not be related at the midpoint of the study, but start to follow the same trajectory from, for example, age 14. We tested this through the correlation between the slope of adolescents’ aggression and the slope of each of the humor types.
- **For H5a,b,c,** we wanted to know if higher levels of preference for television shows featuring disparaging/slapstick/coping humor at the midpoint of the study are related to higher/lower levels of aggression over time. To test this, we examined the correlation between the intercept of disparaging humor preference and the slope of aggression.

**Supplement C**

**Flow Chart of Linear Model Re-Specification Steps**


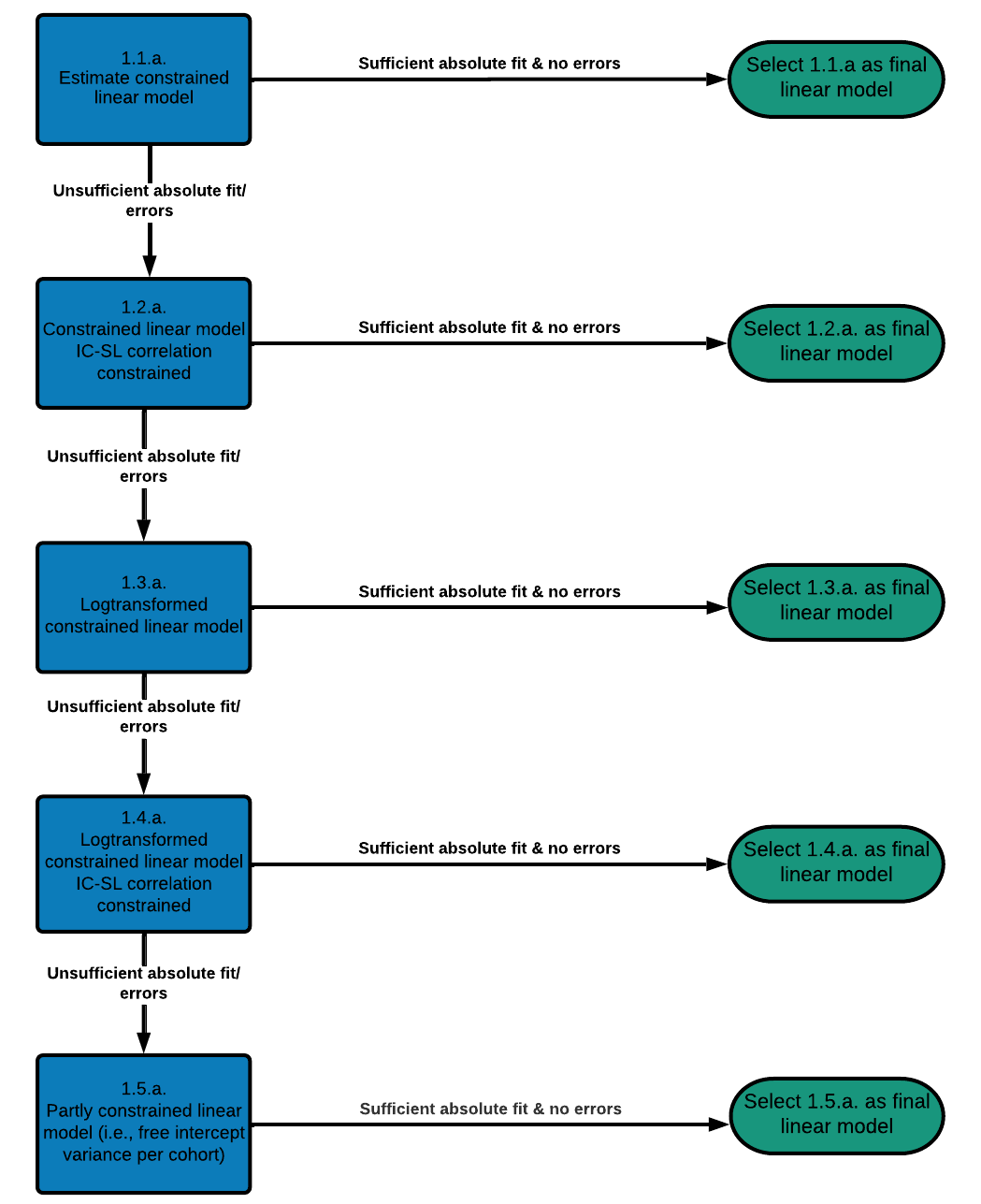


*Note.* See https://doi.org/10.17605/OSF.IO/GA6WC for more information about model comparisons.

**Supplement D**

**Development of Coping Humor With Unconstrained Intercepts for Each Age Cohort**

**Supplement E**

**Graphical Presentation of the Growth Model with Linear and Quadratic Slopes**


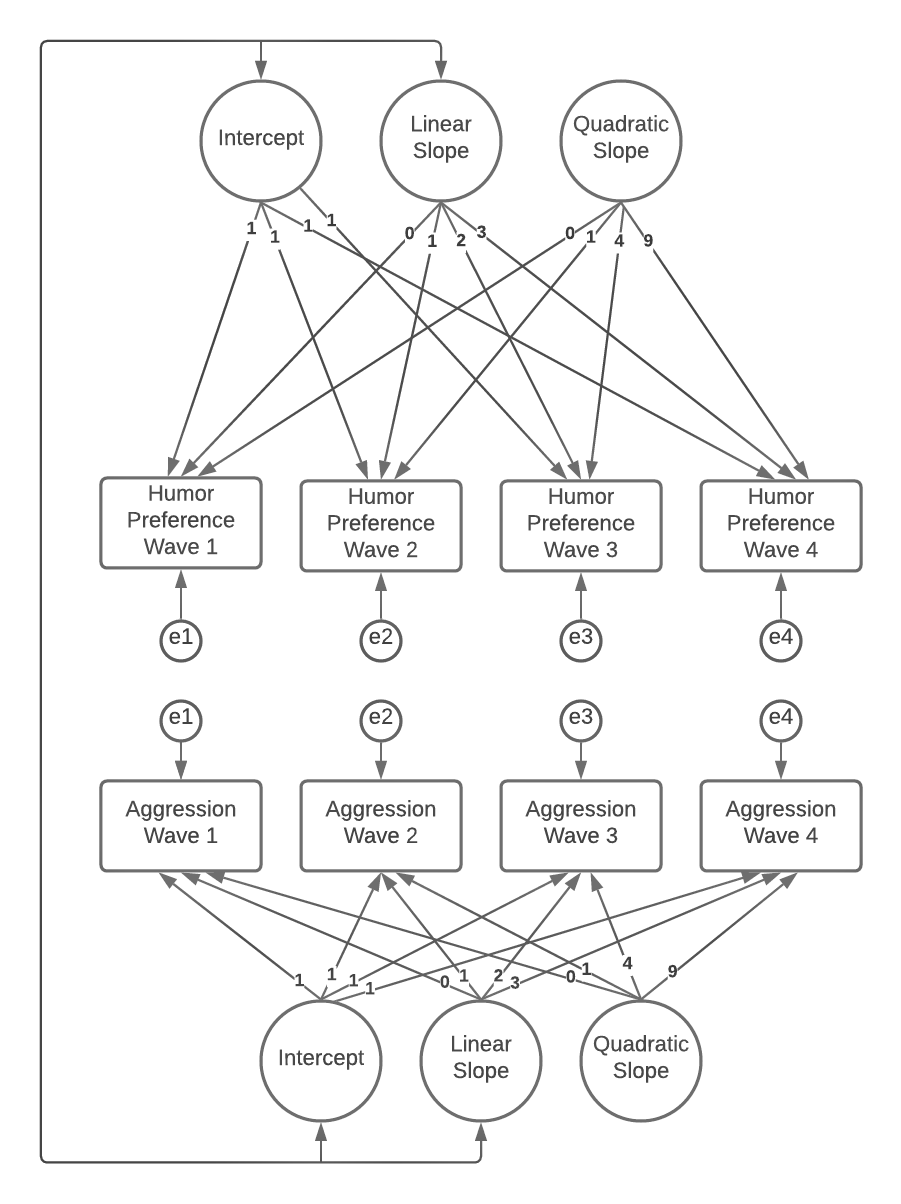


| **Supplement F**  **Means and Standard Deviations at Each Measurement Occasion** | | | | | |
| --- | --- | --- | --- | --- | --- |
| **Variable** | ***N*** | **Min** | **Max** | ***M*** | ***SD*** |
| Aggression Wave 1 | 159 | 1.00 | 4.07 | 1.72 | .70 |
| Aggression Wave 2 | 145 | 1.00 | 3.57 | 1.60 | .57 |
| Aggression Wave 3 | 134 | 1.00 | 3.57 | 1.58 | .56 |
| Aggression Wave 4 | 128 | 1.00 | 3.07 | 1.57 | .50 |
| Disparaging Humor Wave 1 | 165 | .00 | 95.83 | 28.15 | 22.18 |
| Disparaging Humor Wave 2 | 138 | .00 | 93.33 | 26.83 | 22.24 |
| Disparaging Humor Wave 3 | 128 | .00 | 94.59 | 25.42 | 22.14 |
| Disparaging Humor Wave 4 | 124 | .00 | 86.11 | 21.34 | 18.52 |
| Slapstick Humor Wave 1 | 165 | .00 | 78.46 | 15.08 | 15.76 |
| Slapstick Humor Wave 2 | 138 | .00 | 87.10 | 13.43 | 16.46 |
| Slapstick Humor Wave 3 | 128 | .00 | 69.44 | 10.06 | 13.89 |
| Slapstick Humor Wave 4 | 124 | .00 | 86.21 | 8.37 | 12.80 |
| Coping Humor Wave 1 | 165 | .00 | 41.67 | 3.77 | 5.49 |
| Coping Humor Wave 2 | 138 | .00 | 33.33 | 5.28 | 6.54 |
| Coping Humor Wave 3 | 128 | .00 | 53.85 | 5.68 | 7.97 |
| Coping Humor Wave 4 | 124 | .00 | 57.89 | 5.73 | 8.62 |

*Note.* Table reflects untransformed scores. Aggression was measured on a 5-point Likert scale. Humor type preferences are expressed as individual-level proportion scores reflecting the proportion of scenes containing disparaging, slapstick, and coping humor relative to the total number of scenes in that adolescent’s favorite television show(s).
